# The barriers and facilitators to satisfaction with botulinum neurotoxin treatment in people with cervical dystonia: a systematic review

**DOI:** 10.1007/s10072-022-06114-8

**Published:** 2022-05-20

**Authors:** Melani J. Boyce, Alana B. McCambridge, Lynley V. Bradnam, Colleen G. Canning, Arianne P. Verhagen

**Affiliations:** 1 Graduate School of Health, University of Technology Sydney, Sydney, Australia; 2 Physiotherapy Department, Westmead Hospital, Hawkesbury Road, Westmead, Sydney, NSW 2145 Australia; 3 Department of Exercise Sciences, The University of Auckland, Auckland, New Zealand; 4 Faculty of Medicine and Health, The University of Sydney, Sydney, Australia

**Keywords:** Cervical dystonia, Barriers, Facilitators, Botulinum toxin, Satisfaction

## Abstract

**Background:**

Cervical dystonia (CD) is an isolated, focal, idiopathic dystonia affecting the neck and upper back. CD is usually treated by botulinum neurotoxin (BoNT) injections into the dystonic muscles; however, about 20% of people will discontinue BoNT therapy. This systematic review aimed to determine the barriers to satisfaction and facilitators that could improve satisfaction with BoNT therapy for people with CD.

**Methods:**

A database search for journal articles investigating satisfaction with BoNT treatment in CD identified seven qualitative studies and one randomised controlled trial. Results were grouped into “direct” and “indirect” barriers and facilitators.

**Results:**

The most reported direct barrier to satisfaction with BoNT was treatment non-response, reported by up to 66% of participants. Other direct barriers included negative side effects, early wearing-off of treatment effect and inexperience of the treating physician. Indirect barriers included limited accessibility to treatment (including cost) and personal choice. Direct facilitators of satisfaction with BoNT included relief of symptoms and flexible re-treatment intervals. Indirect facilitators included easy accessibility to treatment.

**Conclusions:**

Despite BoNT having a discontinuation rate of only 20%, it appears a much greater proportion of people with CD are dissatisfied with this treatment. As BoNT is currently the main treatment offered to people with CD, efforts to improve treatment response rates, reduce side effects and make treatment more flexible and readily available should be adopted to improve the quality of life for people with CD.

**Supplementary information:**

The online version contains supplementary material available at 10.1007/s10072-022-06114-8.

## Introduction

Cervical dystonia (CD) is defined as an isolated, focal, idiopathic dystonia affecting the neck and upper back [1]. CD presents with life-long motor and non-motor impairments, and currently there is no cure [2]. The primary motor impairment is uncontrollable muscle spasms of the neck; however, at least two thirds of people with CD will also experience long-term non-motor impairments like headaches, neck pain and stiffness [3]. CD causes significant disability affecting work, leisure and social aspects of a person’s life, with 60% of people with dystonia reporting depression and anxiety—a significantly higher rate than the general population [3–5].

The most widely evaluated treatment for CD is botulinum neurotoxin (BoNT) injections into the dystonic muscles. BoNT injections have been shown to improve motor and non-motor impairments, reduce pain and improve quality of life [6, 7]. It is for these reasons that BoNT is the most recommended therapy for CD. However, BoNT therapy is not without its limitations. The injections must be repeated every 3 to 4 months, the injections are painful, and common side effects include dysphagia and excessive muscle weakness [8]. Approximately 20% of people with CD will discontinue BoNT therapy, with reported reasons being a lack of long-term effectiveness or the negative side effects [8]. It is not known whether there are other barriers to the use of BoNT therapy in the CD population, or whether there are facilitators that could improve satisfaction with BoNT therapy for CD.

Therefore, this systematic review aimed to examine current research to address the specific research questions: (1) What are the barriers and facilitators to satisfaction with the use of BoNT treatment in people with CD? and (2) What are the preferences of people with CD who engage in BoNT treatment? The results of this review may help to identify ways to improve the satisfaction with BoNT treatment for people with CD.

## Methods

### Design

We performed a systematic review of the literature, according to the Preferred Items for Reporting Systematic Reviews and Meta-analyses (PRISMA) statement [9]. The review protocol was preregistered on the PRoSPERO database, registration number CRD42019126801.

### Search strategy

Literature searches were developed with the help of a librarian using identified keywords and MESH terms (full search terms shown in supplementary information) from inception to the 4th of August 2021. The following databases were searched: Medline, PubMed, EMBASE, CINAHL and the Cochrane central register of controlled trials. The reference lists of included studies were screened to find additional articles to be included. The search was conducted by the author MB, with the results uploaded onto the Covidence systematic review software (www.covidence.org) for the screening process. Duplicates were removed automatically by the Covidence software.

### Inclusion criteria

Studies were included that investigated adults (18 years or older) with isolated, focal, idiopathic CD. Studies including a mixed dystonia population were also included if the data relating to isolated, focal, idiopathic CD could be extracted. We included peer-reviewed qualitative studies in all languages involving (but not limited to) surveys, interviews, focus groups, or any combination of these methods. All study designs were eligible if they investigated barriers or facilitators to satisfaction with BoNT treatment. Conference abstracts, letters and opinion pieces were excluded.

### Selection process

The titles and abstracts of studies identified by the search were reviewed independently by two out of three authors (MJB, ABM, APV) to determine eligibility. The full text versions of selected articles were independently assessed by the same authors to determine final eligibility. Agreement was achieved by consensus or by the third author (CGC) in case of disagreement.

### Risk of bias assessment

Two reviewers (MJB, LVB) independently assessed each article for risk of bias using The Joanna Briggs Institute Critical Appraisal tool, Checklist for Qualitative Research [10]. Agreement between the reviewers was achieved by consensus and by a third author (CGC) in the case of disagreement.

### Data extraction

Two authors (MJB, LVB) independently performed data extraction. A third author (CGC) adjudicated when there were conflicts. Information on study design, participant details, participant demographics, BoNT delivery and identified barriers and facilitators to satisfaction with BoNT were extracted.

### Data analysis

Qualitative data on barriers and facilitators were collated and pooled into common themes via an iterative discussion with all authors. Identified themes were categorised by being either “direct” or “indirect”. Direct barriers and facilitators were primarily attributable to the effect of the treatment on the person with CD. Indirect barriers and facilitators were related to access, affordability and other issues surrounding BoNT use.

## Results

### Description of included studies

**Fig. 1:**
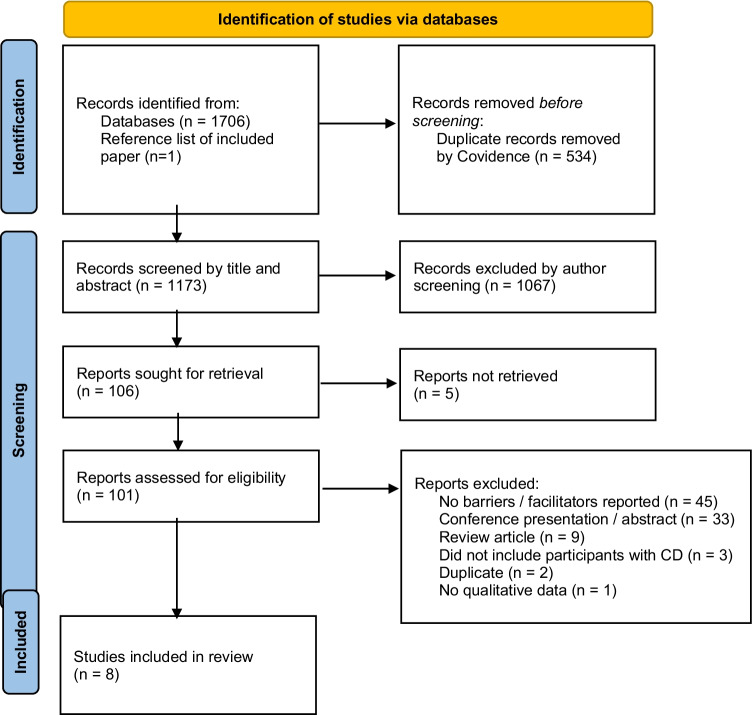
Flow diagram

We found 1707 references with eight studies meeting the inclusion criteria, including 1764 participants in total [3, 11–17] (Fig. 1).

Five studies included only people with isolated focal idiopathic CD [3, 11, 12, 15, 16], two studies included people with CD, blepharospasm and hemifacial dystonia [14, 17] while one study investigated people with Primary CD (isolated, focal, idiopathic CD) and Secondary CD (CD following a neurological event, for example acquired brain injury) [13]. Seven of the studies used qualitative methods, comprising three surveys [3, 11, 15], two medical record file reviews with follow up telephone interviews [12, 14] and two interviews of participants and an online focus group [13, 16]. One randomised controlled trial was included as it used qualitative methodology to examine barriers and facilitators to satisfaction alongside the main aim of evaluating a nurse practitioner led program delivering BoNT to people at home compared to medical practitioner clinic-based delivery of BoNT [17].

One study comprised an international online survey [3], three studies were conducted in the USA [11, 12, 15], two in the UK [16, 17], one in Germany [13] and one in Canada [14]. Eight studies discussed the barriers to the satisfaction of BoNT use [3, 11–17], while five studies discussed the facilitators to the use of BoNT and the preferences of people with CD who engage in BoNT therapy [11, 3, 15–17] (Table 1). Data extracted was categorised into “direct” and “indirect” barriers and facilitators to satisfaction with the use of BoNT as shown in Table 2. “Direct” barriers and facilitators related to the toxin and its delivery while “indirect” barriers and facilitators encompassed factors other than the toxin and its delivery. All qualitative studies were assessed as having a low risk of bias using the Joanna Briggs Institute Critical Appraisal tool, Checklist for Qualitative Research [10].

**Table 1:** Summary of included studies

First author, year	Study characteristics	Barriers (% of responses)	Facilitators (% of responses)
Brashear 2000 [17]	Mailed survey (USA) Population: CD (*n* = 133) % Women: NR Mean age: NR Duration of CD: NR	Dissatisfied BoNT users (*n* = 29): - Non-response *n* = 19/29 (66%) - Side effects *n* = 10/29 (34%) - Lack of access (cost; distance; appointment availability) *n* = 14/29 (48%) - Personal preference *n* = 8/29 (28%) - Other *n* = 7/29 (24%)	- Relief of symptoms: 63% of total population (*n* = 133)
Comella 2015 [3]	Online survey (international) Population: CD (*n* = 1071) % Women: 76% Mean age: 53.2 Duration of CD: 9.6	Dissatisfied BoNT users (*n* = 400) - Non-response (46% of 400) - Side effects (33% of 400) - Early wearing-off of Rx effect (19% of 400) - Physician skill - Lack of access (12% of 400) Non-BoNT users (*n* = 128) - Personal preference (28% of 128) - Lack of access to Rx (14% of 128) - Other (68% of 128) including lack of availability; lack of physician awareness and presence of other dystonias	- Relief of symptoms: 64% of total population (*n* = 1071)
Gill 2013 [11]	File review and telephone interview (USA) Population: CD (*n* = 70) % Women: 77 Mean age: NR Duration of CD: NR	Dissatisfied BoNT users (*n* = 21) - Non-response *n* = 11/21 (52%) - Side effects *n* = 2/21 (10%) - Lack of access (cost; distance) *n* = 7/21 (33%) - Other *n* = 1/21 (5%)	NR
Hausserman 2004 [12]	Face to face or telephone interview (Germany) Population: primary CD (*n* = 79); secondary CD (*n* = 21) % Women primary CD: 59% Mean age: NR Duration of CD: NR	Dissatisfied primary and secondary CD BoNT users (*n* = 33) - Non-response *n* = 4/33 (12%) - Side effects *n* = 11/33 (33%) - Lack of access (cost, inconvenience) *n* = 12/33 (36%)	NR
Hsiung 2002 [13]	Retrospective file review and telephone interview (Canada) Population: CD, blepharospasm, hemifacial spasm, focal dystonia (*n* = 235; CD *n* = 106) % Women CD: 68% Mean age CD: 48 Duration of CD: > 2	Dissatisfied BoNT users (*n* = 49) - Non-response *n* = 20/49 (41%) - Side effects *n* = 3/49 (6%) - Lack of access (cost, inconvenience, distance) *n* = 8/49 (16%)	NR
Jinnah 2016 [14]	Cross-sectional cohort study (USA) Population: CD (*n* = 35) % Women: 74% Mean age: 57.1 Duration of CD: 12.5	- Non-response *n* = 1/35 (3%) - Side effects *n* = 6/35 (17%) - Physician knowledge/skills *n* = 34/35 (97%) - Lack of access (cost) *n* = 1/35 (3%) - Resistance to BoNT *n* = 3/35 (9%)	- Correct treatment dose and injection site relieve symptoms *n* = 25/32 (78%)
Poliziani 2016 [15]	Online focus discussion group and follow up telephone interview (UK) Population: CD (*n* = 31) % Women: 81% Mean age: NR Duration of CD: 16.4	- Side effects - Lack of access (inconvenience, distance to clinic)	- Relief of symptoms - Shorter/flexible injection cycles - More accessible, flexible treatment
Whitaker 2001 [16]	RCT comparing clinic and home-based delivery of BoNT injections (UK) Population: CD, blepharospasm and hemifacial dystonia (*n* = 89; CD *n* = 47) % Women CD: NR Mean age CD: NR Duration of CD: NR	- Non-response - Side effects - Lack of access (inflexible appointment times) - Not enough time with the physician	- Home-based injections (flexible, cost effective and safe service)

Mean age and duration of CD are reported in years

*CD* cervical dystonia, *BoNT* botulinum neurotoxin, *NR* not reported, *Rx* treatment, *RCT* randomised controlled trial

**Table 2:** Categorization of themes of data extraction

Barriers to satisfaction with BoNT	Facilitators of satisfaction with BoNT
*Direct* •Non-response to treatment •Side effects •Early wearing-off of treatment effect (< 3 months) •Knowledge, skill and experience of the treating physician	*Direct* •Relief of symptoms •Flexibility of treatment intervals •Treatment by experienced physician
*Indirect* •Limited access to treatment (including cost, travel, timing, staff shortages, physician and toxin availability) •Personal preference (e.g. dislike of toxin or injection)	*Indirect* •Easy access to treatment and adapting to personal preferences

### Direct barriers

#### Non-response to treatment

Non-response to BoNT treatment was the most reported reason for participants being dissatisfied (six studies) [3, 11–15]. The percentage of people dissatisfied because of non-response or perceived ineffectiveness of BoNT treatment varied between 3 and 66%. In one study, 9% of people were found to have resistance to BoNT, leading to further dissatisfaction with therapy [15].

#### Side effects

The second most common reason for dissatisfaction with BoNT treatment were the side effects, including head drop and dysphagia. Ceasing BoNT due to the side effects occurred in 6–34% of participants in the studies reviewed [3, 11–15].

#### Early wearing-off of treatment effect

Two studies reported participant dissatisfaction with the length of the treatment interval (finding it too long), symptom relief lasting less than three months, the waiting time to access BoNT treatment at the clinic and the inflexible appointment times for re-injection [3, 17]. These were cited by participants as reasons to cease BoNT treatment.

#### Knowledge, skills and experience of the treating physician

One study reported that physician knowledge, skills and experience had an impact on the satisfaction of clients receiving BoNT treatment as 77% of participants were found to have been given an inadequate dose or were injected into incorrect muscles. A further 20% of participants had a type of CD (anterocollis or retrocollis) which was considered difficult to treat effectively by inexperienced physicians [15].

### Indirect barriers

#### Limited access to treatment

Seven papers reported lack of accessibility to BoNT treatment as a barrier to satisfaction [3, 11–16]. Cost or insufficient medical insurance was reported as a barrier to treatment satisfaction in five studies [3, 11–15], while inconvenience and distance to travel to the clinic were reported as barriers in four studies [11–14, 16]. Together, this lack of access to BoNT was reported by between 3 and 48% of participants as reasons for ceasing treatment [3, 11–14, 16]. Finally, a lack of physicians with suitable experience, and subsequent lack of availability of BoNT, were also reported as barriers to satisfaction with treatment in one study [3].

#### Personal preference

Personal choice can be a factor in dissatisfaction with BoNT treatment, usually among those who are non-users of BoNT. Two studies reported dislike of injections or toxin, painful injections and concern about developing antibodies to BoNT as reasons for not using BoNT [3, 11], leading to dissatisfaction with BoNT as a treatment option.

### Direct facilitators

#### Relief of symptoms

The most reported facilitator to the satisfaction with BoNT treatment is the relief of dystonia symptoms. Three studies reported between 63 and 78% of their participants were satisfied with their improved symptoms following treatment, leading to an improved mood, improved confidence, less anxiety when driving and enabling a return to work [3, 11, 15]. When interviewed, people with CD reported they “highly valued any period of relief of symptoms” meaning that the CD had less impact on their quality of life [16].

#### Flexibility of treatment intervals

When interviewed, participants reported that shorter, more flexible, individualised treatment intervals would be preferred as this would offer a more stable relief of symptoms and facilitate satisfaction with BoNT treatment [16].

#### Treatment by an experienced physician

One study investigating people with poor outcomes following BoNT reported that satisfaction could be improved if an experienced physician was delivering the treatment. Experienced physicians were able to choose the correct dose of toxin and location to inject, leading to greater satisfaction with treatment outcomes in 78% of participants [15].

### Indirect facilitators

#### Easy access to treatment and adapting to personal preferences

A randomised controlled trial found that BoNT treatment could be safely delivered to people with dystonia in their homes by trained community-based nurses, with a similar efficacy to clinic-based treatment. This was preferred by participants as it was a more flexible and convenient delivery of treatment, improving the accessibility of BoNT to users and improving satisfaction with treatment [17].

## Discussion

### Main findings

Findings from eight studies indicated the most frequently reported barriers to satisfaction with BoNT treatment in people with CD are non-response to treatment, the treatment effect wearing off before the next scheduled injection, and the negative side effects. Less common barriers were the limited access to treatment in terms of location of experienced physicians, cost and flexibility of treatment times. Facilitators to the satisfaction with BoNT treatment were reported as treatment effectiveness, ease of access and flexibility of BoNT delivery and treatment interval.

### Comparison with other studies

Similar findings have been reported in two studies of different neurological populations accessing BoNT in the treatment of spasticity [18, 19]. One study investigated the reasons why people with spasticity from multiple sclerosis discontinued their BoNT treatment and found that loss of efficacy was the most reported reason for ceasing BoNT, followed by logistical problems accessing the clinic and adverse events following treatment [18]. A survey of Australian neurological physiotherapists and occupational therapists reported that the barriers to BoNT treatment for people with spasticity from various neurological conditions were the financial cost of BoNT for patients and access to a specialised spasticity clinic [19]. Waiting times to be assessed at a spasticity clinic were also reported as a barrier to treatment, indicating a lack of appropriately qualified physicians to service the needs of the community.

### Strengths and limitations

A strength of this review is that it synthesises qualitative studies in people with CD, which is novel for this population. Qualitative studies support a patient-centred approach to treatment and may assist in highlighting ways to adapt practices to improve the patient experience of treatment. Another strength of this review is the fact that participants of individual studies came from five continents. Most of the studies presented were conducted in Europe or North America; however, one large-scale international survey [3] reported responses from people with CD from Europe, North and South America, Australia, New Zealand, Africa and Asia, giving a global view of the perspectives of people with CD. Therefore, the results can be translated to people with CD globally.

The limitations of this review were the relatively small number of studies included and the small sample sizes of most studies, likely owing to the rarity of the condition. Considering BoNT is the primary treatment option for people with CD, this review highlights the lack of published qualitative research related to patient satisfaction with BoNT and the preferences of people with CD. It is likely that the barriers to BoNT use reported here contribute to the treatment discontinuation rate of approximately 20% among people with CD [8].

### Future directions

As CD presents differently for each person, adopting a “precision medicine” approach to management could add to satisfaction with treatment [20]. This approach has been adopted in the diagnosis and management of various long term neurological conditions including stroke, Parkinson’s disease and other types of dystonia [21–23]. Precision medicine aims to target treatment to the individual’s genetic, biomarker, phenotypic or psychosocial characteristics in order to provide an individual, specific therapy for that person [20]. If this can be achieved for people with CD, the outcome would likely provide the most satisfaction with BoNT treatment.

Some aspects of BoNT treatment for people with CD could be easily individualised to improve satisfaction, specifically the flexibility of dosage, treatment intervals and delivery of BoNT therapy. Commonly, dosage of BoNT for CD is individualised however frequency of delivery is not. Our review has shown that people with CD would prefer treatment intervals that are more flexible and self-determined to improve their satisfaction with treatment. Difficulty with accessing skilled physicians was another reported barrier to treatment satisfaction which could be addressed by providing formal training programs for physicians administering BoNT in order to staff more specialised clinics and ensure a standardised level of knowledge and skill in treating physicians. Innovative models of delivery of BoNT could also improve access to treatment, as seen in a randomised controlled trial of community nurse BoNT delivery [17]. This novel model of BoNT delivery was cost effective and preferred by participants.

Another barrier to satisfaction found in our review was the side effects. Common side effects of BoNT treatment include swallowing problems, excessive neck weakness, pain, muscle atrophy, generalised malaise and head drop [11, 14, 15], with a higher risk of side effects found when a medium or high dose of toxin is injected [24]. It follows that minimisation of side effects might be achieved by utilising the lowest possible effective dose of toxin into the correct muscles. One study found that re-treatment by experienced physicians improved the outcomes for 78% of participants who had previously been unsatisfied with their treatment [15], indicating that access to specialised physicians for treatment is an important factor in improving satisfaction with BoNT.

Overall, a precision medicine approach with flexible delivery of treatment by specialised physicians would ideally result in the optimal treatment effect and therefore improved satisfaction with BoNT for people with CD.

### Future research

Although BoNT treatment has been shown to be effective in reducing pain and improving quality of life [6, 7], one of the main barriers is perceived non-effectiveness. Apparently, a significant proportion of patients do not experience BoNT as effective despite the evidence. Future research should evaluate whether this is due to the heterogenous nature of dystonia displayed by patients participating in the trials, the selected outcome measure, or the difference in BoNT protocols used in trials compared to daily practice. Investigating the reasons why some people do not respond to BoNT treatment is also important. Future studies could also investigate the effect of a precision medicine approach on treatment effectiveness and the satisfaction of people with CD.

In addition, further research should investigate the optimal BoNT treatment interval for people with CD. Currently the recommended treatment interval is 10–12 weeks (to reduce the risk of developing secondary resistance to the drug), however up to 45% of people find this interval too long [7]. In a study of flexible treatment intervals determined by participants, intervals as short as 6 weeks were found to be safe [25]. Satisfaction with treatment and treatment effectiveness would likely be improved for people with CD by self-determining their treatment intervals if it is safe for them to do so.

## Conclusion

We found that common barriers to satisfaction of BoNT for people with CD are poor perceived effect of the treatment, poor sustainability of the treatment effect and the negative side effects. Cost and accessibility of treatment are also barriers for some people. To facilitate increased satisfaction with BoNT treatment, it is suggested that a precision medicine approach should be adopted to enable individualised treatment dosage and intervals of BoNT to improve treatment effect. Ease of access to skilled physicians and minimising cost is also important to enable people with CD to be most satisfied with their treatment.

## Supplementary Information

Below is the link to the electronic supplementary material.

Supplementary file1 (DOCX 16 KB)

## Data Availability

The data that support the findings of this study are available from the corresponding author upon reasonable request.
