# Supplementary material for: The barriers and facilitators to satisfaction with botulinum neurotoxin treatment in people with cervical dystonia: a systematic review
Source: Neurol Sci. 2022 May 20;43(8):4663–70. doi: 10.1007/s10072-022-06114-8 (PMC9349125; doi:10.1007/s10072-022-06114-8)
Supplement: Supplementary file 1 — Supplementary file1 (DOCX 16 KB) [file 10072_2022_6114_MOESM1_ESM.docx]

**The barriers and facilitators to satisfaction with botulinum neurotoxin treatment in people with cervical dystonia: a systematic review.**

**Authors:**

Boyce MJ^1,2^, McCambridge AB^1^, Bradnam L^3^, Canning CG^4^, Verhagen AP^1^

**Affiliations:**

1 Graduate School of Health, University of Technology Sydney, Australia

2 Physiotherapy Department, Westmead Hospital, Sydney, Australia

3 Department of Exercise Sciences, The University of Auckland, Auckland, New Zealand

4 Faculty of Health Sciences, The University of Sydney, Sydney, Australia

**Corresponding Author:**

Melani Boyce

Email: [Melani.boyce@health.nsw.gov.au](mailto:Melani.boyce@health.nsw.gov.au)

**Supplementary information:**

Search terms – keywords and MESH terms

The keywords and MESH terms used in the search were as follows:

“Exploratory qualitative research” OR “health survey” OR “patient health questionnaire” OR “questionnaire” OR “qualitative research” OR “survey” OR “quality of life” OR “patient reported” OR “satisfaction” OR “patient satisfaction” OR “life satisfaction”;

AND

“Botulinum” OR “Botulinum toxin A” OR “Botulinum toxin” OR “BTX” OR “Botulinum toxin B” OR Dysport OR “Neurotoxin” OR “muscle relaxant agent”;

AND

“dystonia” OR “torticollis” OR “dystonic disorders” OR “focal hand dystonia” OR “paroxysmal dystonia” OR “segmental dystonia” OR “multifocal dystonia” OR “myoclonus dystonia” OR “generalised dystonia” OR “musician’s dystonia” OR “torsion dystonia” OR “cervical dystonia” OR “oromandibular dystonia” OR “focal dystonia”.
